# Proximity-Assisted Synthesis of Large Area MoS_2_ on Different Target Substrates by Chemical Vapor Deposition Using a Mo Nanofilm Substrate

**DOI:** 10.3390/nano16030159

**Published:** 2026-01-24

**Authors:** Muhammad Tariq, William Poston, Norah Aldosari, Gregory Jensen, Maryam Bizhani, Eric Stinaff

**Affiliations:** 1 Department of Physics and Astronomy, Ohio University, Athens, OH 45701, USA; mt967721@ohio.edu (M.T.); wp826418@ohio.edu (W.P.); na314617@ohio.edu (N.A.); gj772812@ohio.edu (G.J.); mb795116@ohio.edu (M.B.); 2 Nanoscale and Quantum Phenomena Institute (NQPI), Athens, OH 45701, USA; 3 Department of Physics and Astronomy, College of Science and Humanities, Prince Sattam bin Abdulaziz University, 173, Al-Kharj 16278, Saudi Arabia

**Keywords:** transition metal dichalcogenide, Mo film, CVD, sulfur, proximity, Raman, photoluminescence

## Abstract

Despite efforts to produce scalable, substrate-independent, low-defect-density, and high-quality MoS_2_, this continues to be a critical challenge for industrial-scale applications. This work aims to present a chemical vapor deposition (CVD) method for growing high-quality and potentially large-area mono- to few-layer MoS_2_ films via proximity between the Mo nanofilm substrate and the target substrates. By using stoichiometry-guided knowledge of Mo-S and Mo-O-S phase diagrams, Mo nanofilms are oxidized and then sulfurized under optimized conditions to grow high-quality, millimeter-scale mono- to few-layer MoS_2_ films in proximity to the target substrate. We have achieved millimeter-scale continuous growth of MoS_2_ revealed via optical microscopy. Two-dimensional Raman maps of Full Width at Half Maximum show high-quality growth, and photoluminescence-based B/A exciton amplitude ratio shows high crystalline and optical quality with low defect density.

## 1. Introduction

Scientific research on two-dimensional (2D) materials has discovered diverse groups of layered structures with remarkable physical properties. Transition metal dichalcogenides (TMDs) have appeared as a varied and rich family within this group of 2D materials because of their semiconducting properties [1]. TMDs are represented by the general formulae MX_2_, where M is the transition metal that belongs to groups 4 through 10 of the periodic table, and X is a chalcogen that belongs to group 16. The chemical composition, crystal structure, and quantum confinement in TMDs give rise to peculiar behavior in their physical properties. Each monolayer TMD holds a layer of transition metal (M) atoms sandwiched between two layers of chalcogen (X) atoms to form a tri-layered atomic plane [2]. The monolayers, each with a thickness of ~0.65 nm, are stacked together by weak van der Waals forces to form bulk materials [3]. MoS_2_ and WS_2_ are members of the TMDs, which change from an indirect bandgap to a direct bandgap from bulk to monolayer form. These are well known as quantum materials, which are incredibly useful for futuristic devices, for example, in the fields of optoelectronics, flextronics, photonics, and quantum information technologies [4,5,6].

The research for scalable, uniform, continuous, reproducible, and high-quality synthesis techniques of TMDs like MoS_2_ and WS_2_ is the primary focus of researchers for its use in semiconducting devices to transform from the basic research facility to large-scale fabrication. Among synthesis techniques, chemical vapor deposition (CVD) is the most widely used bottom-up approach for producing high-quality and large-scale TMD films [4,5,6]. However, it faces challenges of uniform growth, defect density control, and substrate-independent growth. These limitations can be overcome using Plasma Enhanced CVD (PECVD), pulsed CVD, and strategies like thermal mismatch, substrate engineering, and space confinement [6,7,8,9]. For example, space confined setup combined with ambient pressure CVD has yielded a 2-inch WS_2_ film to control layer thickness and modulate nucleation density for use in advanced optoelectronic applications [7]. Similarly, innovative research showed the growth of wrinkled WS_2_ nanostructures using a thermal mismatch strategy for potential use in strain sensing and non-linear optics applications [8]. Emerging CVD techniques have achieved lower growth temperatures, uniformity, control of defects, fine-tuning of composition, modulation of growth kinetics, and improved material properties [6]. In addition, precursor engineering and two-step conversion techniques improved crystallinity and stoichiometry [10,11].

For the growth of MoS_2_ using CVD, compounds having molybdenum (Mo) and sulfur (S) precursors chemically react at elevated temperatures in the presence of an inert gas, such as argon (Ar), to grow MoS_2_ on a substrate. Precursors, such as oxides of Mo and S containing compounds, are managed to react chemically in a reaction chamber called the growth zone, allowing MoS_2_ to grow on a substrate. At the growth temperature, sulfur reduces oxides of Mo to synthesize MoS_2_. For example, a research team from the University of Oxford described the shape evolution of monolayer MoS_2_ crystals using the CVD method and the effect of variation of molybdenum and sulfur precursor concentrations [12]. They investigated the impact of Mo ratios on different crystal morphologies and the contribution of the temperature gradient along the dual furnace CVD setup. An alternative approach by Chang et al. (2020) was focused on the importance of the design of the precursors and experiments to control the lateral growth and how it enhances lateral growth by self-limiting reaction to achieve continuous and uniform 2D films [13]. A pioneering approach by Choi et al. (2020) showed the proximity technique for growing inch-scale 2D MoS_2_ films using two Mo films on sapphire substrates [14]. The study saw how Mo films on two sapphire substrates form self-limiting oxides, which are then sulfurized using H_2_S to form a uniform and continuous monolayer of MoS_2_. The results showed a uniform and single-crystal monolayer by using physical characterization techniques [14]. Their work provides optimized parameters for the process, which helps to achieve a large-area and uniform monolayer of MoS_2_. A similar two-step approach has also been shown by another group without using toxic H_2_S gas for growing MoS_2_ [15]. However, they grew only on sapphire substrates, which works well for the proof-of-concept growth process but could be challenging and costly for integration into industrial processes. We provide a qualitative and quantitative comparison of our proximity-assisted CVD technique with the previously reported similar techniques in Table 1. As the substrates like sapphire are costly, we achieved high-quality mono- to few-layer growth on different substrates and achieved growth on both substrates, making our technique cost-effective compared to others.

Taken together, these studies suggest that the growth using the CVD techniques depends on the types of precursors, growth temperature, type of carrier gas, and its pressure; however, synthesizing a uniform, high-quality, continuous, and large-area monolayer MoS_2_ film on a variety of substrates is still a critical challenge. Here, we present an unconventional and new growth technique for two-dimensional (2D) molybdenum disulfide (MoS_2_) nanofilms using the ambient pressure CVD method via proximity between a Mo film on SiO_2_/Si substrate and other substrates such as SiO_2_/Si, sapphire, and Si. Our research investigates a CVD method, which offers an adaptable technique for synthesizing large-area, uniform, and high-quality 2D MoS_2_ films. We used stoichiometric knowledge guided by phase diagrams to develop a new proximity technique for the growth of MoS_2_. This research aims to set up a potentially large-area mono- to few-layer of MoS_2_ on distinct types of substrates compatible with industrial processes. The findings show the growth method provides a promising pathway toward scalable synthesis of MoS_2_ with some level of control over uniformity and crystallinity.

## 2. Materials and Methods

**Figure 1:**
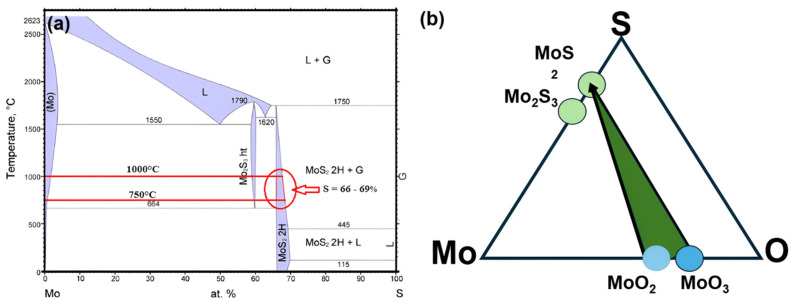
(**a**) Binary phase diagram of the Mo-S system [16], (**b**) ternary phase diagram of the Mo-O-S system showing pathways [17].

The synthesis of MoS_2_ with desired properties is affected by the stoichiometry of precursors, explained by the Mo-S and Mo-O-S systems phase diagrams. Consideration of stoichiometry and these diagrams is fundamental for manipulating growth to achieve the intended material properties. The Mo-S binary phase diagram displayed in Figure 1a shows the difficulty of the chemical reaction between Mo and S to synthesize pure MoS_2_ and specific sulfide phases of interest. It shows that stable sulfides, i.e., MoS_2_ and Mo_2_S_3_, can be grown from their elements under specific conditions of temperature, pressure, and precursor concentrations. Mo_2_S_3_ is stable just above 610 °C, and MoS_2_ exhibits a stable 2H form in a wide range of temperatures. In the range of growth temperatures from 750 °C to 1000 °C, the sulfur content in MoS_2_ lies between 66% and 69% and presents difficulties in keeping stoichiometry during MoS_2_ synthesis. The area of the 2H region, as highlighted in the diagram, focuses on the challenge of supporting reliable precursor ratios. The slight variations in these parameters can change the sulfur content and influence the quality of the grown material. Higher sulfur concentrations can lead to the formation of amorphous forms and hinder attempts to synthesize uniform MoS_2_ [16].

The ternary phase diagram is a triangle with Mo, O, and S at the vertices, illustrating the coexistence of distinct phases in a green triangle, i.e., MoO_2_, MoO_3_, and MoS_2_, as shown in Figure 1b. The Mo-O-S diagram provides insight into the synthesis of MoS_2_ using CVD techniques. These diagrams can help to envision the reaction pathways and challenges involved. Sulfurization of molybdenum thin films is one of the widely researched key techniques for growing MoS_2_, but it can also lead to unwanted phases, such as Mo_2_S_3_. Additionally, the sulfurization of molybdenum’s stable oxides, specifically MoO_2_ or MoO_3_, has been employed in CVD techniques for the growth of MoS_2_. In our growth technique, we propose the following possible reaction pathways for the development of MoS_2_ films [17].

Mo + 2S → MoS_2_ (1)

2MoO_3_ + 4S → 2MoS_2_ + 3O_2_ (2)

MoO_2_ + 2S→MoS_2_ + O_2_ (3)

Phase diagrams help predict the challenges of MoS_2_ synthesis, such as sulfur concentration, temperature, and growth time, to limit the formation of undesirable phases. Changes in these parameters can alter the growth process and lead to deformities that compromise the material’s quality. The coexistence of MoS_2_, MoO_2_, and MoO_3_ requires reasonable control of these parameters to complete the reaction. These phase diagrams function as a roadmap for predicting and optimizing the growth of high-quality MoS_2_.

**Figure 2:**
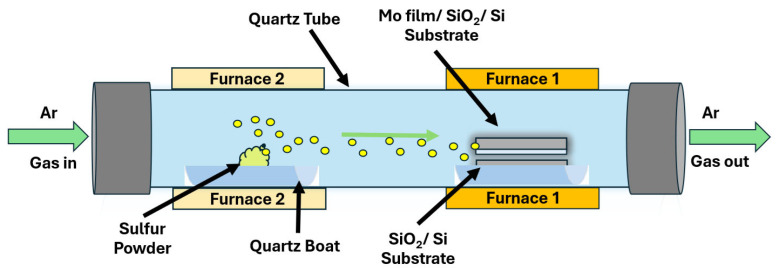
Chemical vapor deposition (CVD) setup—double furnace system.

A conventional double-furnace CVD system consists of a 1-inch-diameter quartz tube and is used for the growth of MoS_2_, as displayed in Figure 2. This new growth technique includes the following four fundamental steps: a warm-up step, an oxidation step, a growth step, and a cooldown step. For the experimental setup, a clean substrate is situated face-to-face below a molybdenum (Mo) thin film-coated substrate; complete sputtering details are provided in the Supplementary Materials. A gap of 0.5–1 mm is supported between the two substrates using a curved quartz boat piece as a sample holder. The sample holder is then carefully positioned in the central region of the growth furnace. At the same time, the second furnace is used to hold a quartz boat holding 10–20 milligrams of precursor sulfur powder. This boat is situated at the midpoint of the second furnace. After the apparatus is arranged correctly, the quartz tube is securely fixed with accessories, and Ar gas is allowed to flow as the carrier gas for sulfur. The gas passes through the quartz tube in a manner that the growth substrate is positioned downstream of the sulfur precursor, as shown in Figure 2.

**Figure 3:**
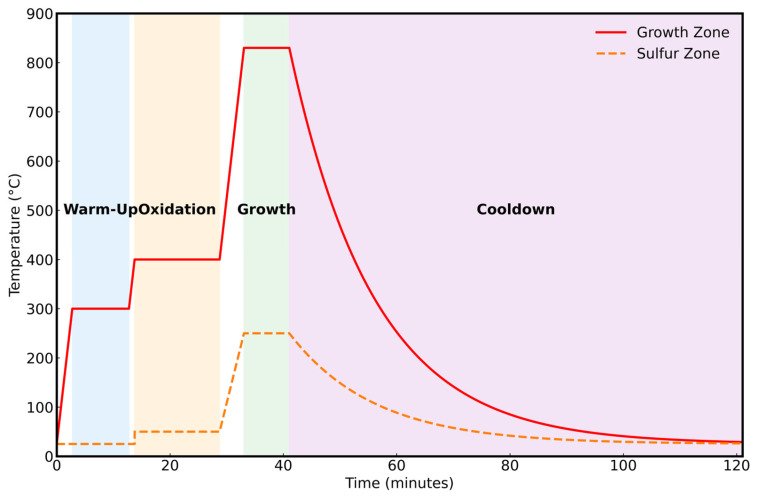
A general temperature profile of the process.

During the first stage, Ar gas is made to flow at a rate of 15 sccm to flush and purge the quartz tube, minimizing the contamination. The growth furnace is kept between 250 and 300 °C to drive off adsorbed moisture from the growth zone, while the precursor furnace is kept at room temperature. This step lasts approximately 5–10 min, keeping the Ar gas flow rate the same. In the oxidation step, the temperature of the growth region is raised to 350–450 °C to perform oxidation of the Mo film with residual (background) oxygen present in the tube while Ar flows at a rate of 15 sccm through the quartz tube. Simultaneously, the sulfur precursor furnace is warmed to 50 °C to remove moisture from the powder and prepare a dry sulfur source. This step typically lasts 10–15 min to achieve the best oxidation. For the growth step, the Ar gas flow rate is reduced to 5 sccm, and the temperature in the growth zone is increased to 780–860 °C, depending on the thickness of the Mo film. As the temperature approaches the intended growth temperature, the sulfur zone is heated up to 250 °C to vaporize the sulfur and allow it to flow into the growth zone. This step lasts 5–8 min to achieve a reaction between the sulfur fumes and the oxides of Mo, leading to the growth of a mono- to few-layer MoS_2_ on the bottom substrate. A general temperature profile of the steps is shown in Figure 3. The furnaces are then set to cool down to room temperature under a consistent flow of Ar gas after the growth step.

## 3. Results and Discussion

**Figure 4:**
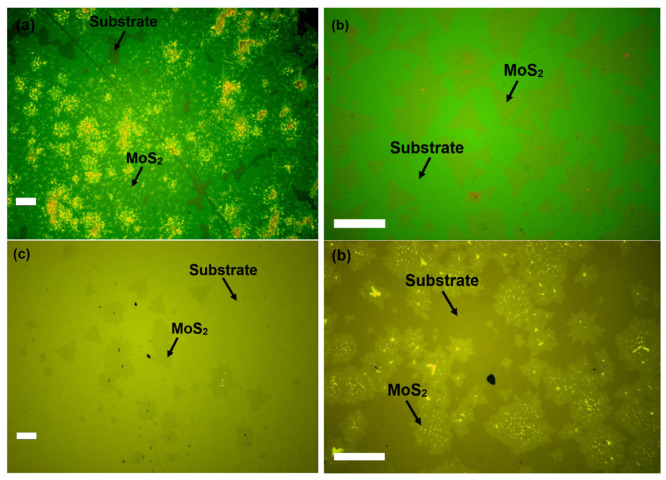
(**a**) As-grown MoS_2_ on wet oxide 300 nm SiO_2_/Si, (**b**) As-grown MoS_2_ on dry oxide 800 nm SiO_2_/Si, (**c**) As-grown MoS_2_ on Si, (**d**) As-grown MoS_2_ on sapphire (scalebars at 100 µm).

Multiple carefully designed growth experiments were performed to achieve the reproducible synthesis of mono- to few-layer MoS_2_ using the proximity technique at ambient pressure. We used wet oxide 300 nm SiO_2_/Si, dry oxide 800 nm SiO_2_/Si, Si, and sapphire substrates for the growth of MoS_2_ in the series of experiments, as shown in Figure 4a–d. For the growth of MoS_2_ on sapphire, we used miscut c-plane sapphire using this technique, to compare with reports of wafer-scale single-crystal growth on such substrates [18]. The annealing details are provided in the Supplementary Materials. It is also relevant to note that graphite boats were used in the first experiments. However, their tendency to cause carbon contamination and uncontrolled nucleation, along with bulky non-stoichiometric MoS_2_ films as discussed in the Supplementary Materials, compelled the use of quartz boats. This strategy was also helpful in keeping separation between substrates by using quartz boat curvature to create a vertical gap between the Mo film substrate and the growth substrate. The use of the quartz boat also helps in keeping the Mo:S ratio because of no sulfur consumption by the boat and yields with low defects or carbon doping as revealed by the Raman peaks FWHM values and PL intensity maps as discussed below.

**Figure 5:**
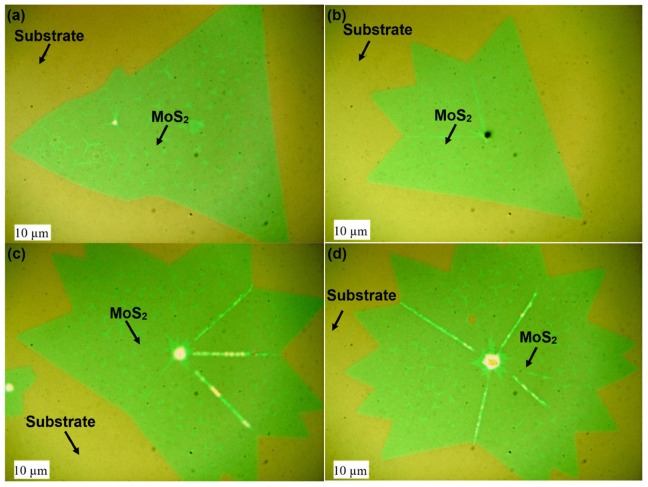
(**a**–**d**) As-grown MoS_2_ individual flakes on wet oxide 300 nm SiO_2_/Si substrate.

The optical microscopy images in Figure 5 show as-grown individual MoS_2_ flakes using proximity technique. The size of individual flakes of MoS_2_ growth was seen on the millimeter (mm) scale, and the size of continual growth was on the centimeter (cm) scale, images are provided in Supplementary Materials. The as-grown MoS_2_ showed high-quality uniformity over an investigated area, with random nucleation sites of bulk material remaining. Though the triangle is the most common shape of MoS_2_ growth, flakes with unusual shapes were noticed. These variations in shape can be attributed to the self-limiting Mo: S precursor ratios on the SiO_2_ surface [14,19]. During the growth process, thermal displacement of the Mo oxide film by S atoms in very close proximity to the growth substrate can create concentration gradients and random patterns, giving rise to unusual growth.

The theory of crystal growth suggests that the morphology of crystals is regulated by the relative growth rates of their crystal faces, starting at the nucleation sites. The slow-developing features govern the final shape, while quickly developing sides vanish or fade out. The growth rate is attributed to the surface free energy and low-energy features developing more slowly than high-energy faces, allowing them to become prominent [12]. The shape of the monolayer MoS_2_ is decided by the growth rate of the terminating features. The Mo nanofilm also influences crystal morphology, and any change in the Mo:S ratio can affect the chemical activity of the terminating faces. The theoretical dynamics of crystal growth suggest that it is hard to achieve precise control over the growth process without controlling the crystal seeds or nucleation sites [20,21,22,23].

The investigations focused on studying and optimizing the effects of growth parameters, including temperature within the experimental range of 780 °C to 860 °C, precursor content from 10 to 30 mg, separation between substrates within a range of ~0.4 to 1.7 mm, and thickness of the Mo film for sputtering time 1 to 10 min. The substrates, with a maximum area of 1 inch × 1/2 inch, were used for the series of experiments due to the diameter limitation of a 1-inch quartz tube setup. However, this technique can be scaled up for further possible large-area growth. With carefully controlled conditions, the proximity growth technique produced high-quality and reproducible mm-scale mono- to few-layer MoS_2_ and individual flakes of MoS_2_ monolayers. Optimal conditions were achieved by adjusting parameters such as temperature, substrate distance, and Mo film thickness. This technique requires reasonable control to support reproducibility, as any variation can result in unusual growth patterns that conflict with earlier reported findings. A temperature of ~820 °C was identified as favorable for growing high-quality mono- to few-layer MoS_2_, using ~60 nm Mo film substrate under controlled conditions. Additionally, the distance between the substrates of ~0.5 mm was found to be crucial for consistent growth results. As the growth temperature is the main factor for the structure and morphology of as-grown MoS_2_, we performed multiple experiments using a thickness of less than 100 nm at different reaction temperatures to find the optimal growth temperature. Later, we fixed the range of ~15–60 nm Mo film thickness as this was producing growth on both the bottom, clean substrate, as well as the top substrate with the Mo film, making our experiments cost-effective. Optimal temperatures were found empirically, based on the coverage (≥70%) of MoS_2_ growth on the sample. The optimal growth temperature values of 780 °C, 800 °C, and 820 °C correspond to Mo film thicknesses of 15 nm, 40 nm, and 60 nm, respectively, for improved crystallinity and quality of MoS_2_ growth. It was seen that an increase of 10 nm thickness requires ~15 to 20 °C to support the thermal vaporization and mass transfer of oxides of Mo.

To confirm our results and assess the quality of the as-grown material on different substrates, we conducted Raman and photoluminescence (PL) spectroscopy on as-grown MoS_2_ using a 532 nm excitation laser, as shown in Figure S4. Both Raman and PL spectroscopy are sensitive to the number of layers as well as the quality of the material and are therefore two of the most extensively used characterization techniques. For Raman spectroscopy, the E^1^_2g_ mode is associated with the in-plane vibration of Mo atoms in one direction and S atoms in the opposite direction, respectively, while the A_1g_ mode corresponds to the out-of-plane vibration of S atoms in opposite directions. The wavenumber difference between the two vibrational modes is characteristic of the number of layers with bulk material having a separation of ~26 cm^−1,^ which decreases to ~19 cm^−1^ for a monolayer [24]. Additionally, it has been shown that the defect density in the as-grown MoS_2_ causes the peak broadening, and the Full Width at half maximum (FWHM) of the Raman modes is related to the quality of the 2D MoS_2_ [25,26]. Specifically, earlier work reported that the FWHM of the E^1^_2g_ mode is a quantitative indicator of defect density in the monolayer of MoS_2_, which was grown by the CVD method on SiO_2_/Si substrate. They correlated the narrow FWHM to the low defect density (~10^12^/cm^−2^) and classified growth as a high-quality monolayer. For PL, multilayer MoS_2_ has an indirect bandgap (~1.2–1.3 eV) and shows reduced PL intensity by orders of magnitude due to the indirect transition. This weak or negligible emission with broadened width is due to the low quantum efficiency of phonon-assisted transitions [24,25]. In contrast, monolayer MoS_2_ has a direct bandgap (~1.8–1.9 eV) and shows strong PL intensity with quantum yield orders of magnitude higher due to direct optical transitions dominated by A exciton peak and often with weak B exciton peak [24,25]. 

As a qualitative measure of defect density, McCreary et al. (2018) showed that the B/A intensity ratio could show the presence of defects in the as-grown material, and a high ratio corresponds to high defect density [27,28,29]. It is important to note that the PL B/A ratio can also be influenced by factors like strain, substrate-induced charge transfer, and dielectric effects. These factors can affect the excitonic recombination process and change the bandgap, useful for bandgap and strain engineering-based technologies [30]. Also, interaction with the target substrate changes the electronic states of MoS_2_ and causes changes in excitonic behavior [31]. Additionally, Hossen et al. (2025) studied that the growth with lower defect density shows higher PL intensity and vice versa, which is attributed to variation in the pristine band structure of MoS_2_ [25]. Below, we present spatially resolved maps of the Raman and PL spectra for as-grown material on SiO_2_/Si, sapphire, and Si. We found a Lorentzian to provide the best fit for evaluating the Raman spectra, indicating that the dominant component was from the intrinsic, homogeneous broadening. For the photoluminescence (PL) spectra, we found a Gaussian profile to provide the best fit, indicative of the typical inhomogeneous broadening observed in room-temperature PL from MoS_2_ [32,33].

**Figure 6:**
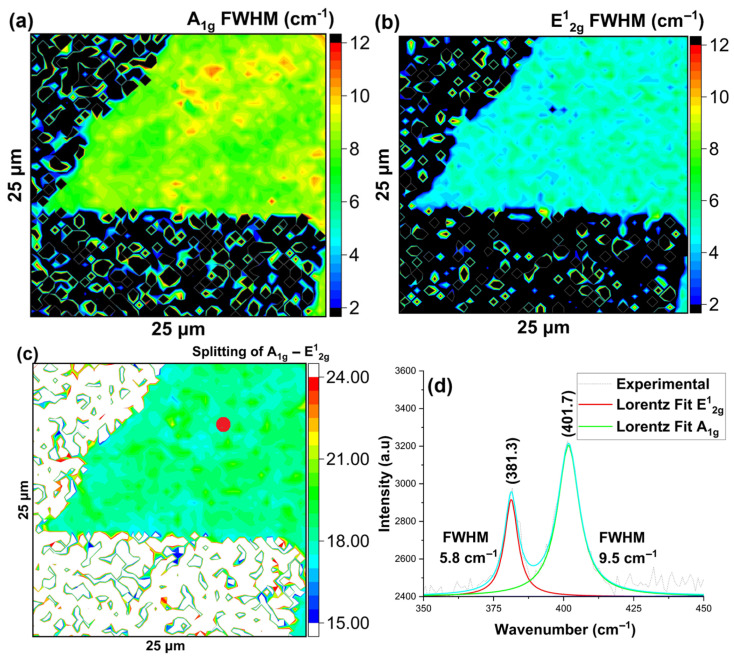
MoS_2_ growth on SiO_2_/Si substrate using quartz boat (**a**) Map of A_1g_ Raman Mode’s Full Width at Half Maxima (FWHM), (**b**) Map of E^1^_2g_ Raman Mode’s Full Width at Half Maxima (FWHM), (**c**) Map of Raman band Splitting of A_1g_ and E^1^_2g_ modes, (**d**) Representative Raman spectra of the indicated (red) spot. The blue line represents the cumulative fit.

**Figure 7:**
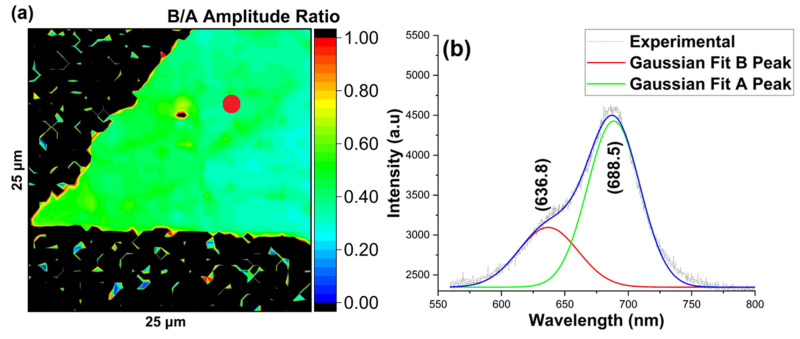
MoS_2_ growth on SiO_2_/Si substrate using quartz boat (**a**) map of B/A amplitude ratio obtained by Gaussian fitting of PL intensity map, (**b**) representative PL spectra of the shown (red) spot. The blue line represents the cumulative fit.

First, for material grown on SiO_2_/Si substrates, the FWHM maps of the A_1g_ and E^1^_2g_ Raman modes revealed the uniformity and high quality of the monolayer over the investigated area, as shown in Figure 6a,b. The splitting between A_1g_ and E^1^_2g_ Raman modes, as shown in Figure 6c,d, indicates the presence of a monolayer, while the B/A ratio of the Gaussian amplitudes and PL intensity suggested the presence of low defects related to impurity or vacancy-induced localized states, as shown in Figure 7a,b.

**Figure 8:**
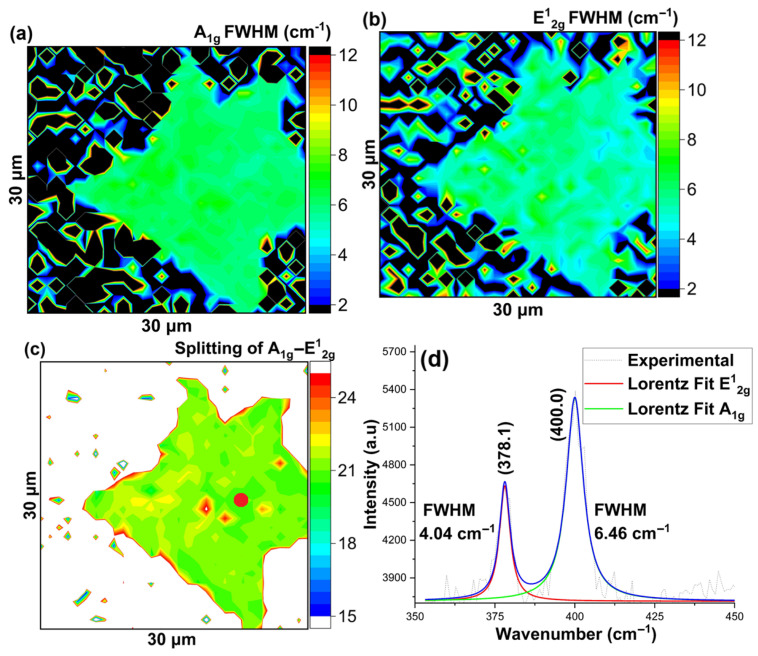
MoS_2_ growth on sapphire substrate using quartz boat (**a**) Map of A_1g_ Raman Mode’s Full Width at Half Maxima (FWHM), (**b**) Map of E^1^_2g_ Raman Mode’s Full Width at Half Maxima (FWHM), (**c**) Map of Raman band Splitting of A_1g_ and E^1^_2g_ modes, (**d**) Representative Raman spectra of the indicated (red) spot. The blue line represents the cumulative fit.

Next, we show Raman and PL spectroscopy of a MoS_2_ flake grown on the sapphire substrate. We took 2D spatial maps of Raman modes and PL intensity by integrating the spectra over an area of 30 μm × 30 μm of a MoS_2_ flake. Similar to the SiO_2_/Si substrate, the FWHM maps of the Raman modes showed the excellent quality of the MoS_2_ flake, as shown in Figure 8a–d.

**Figure 9:**
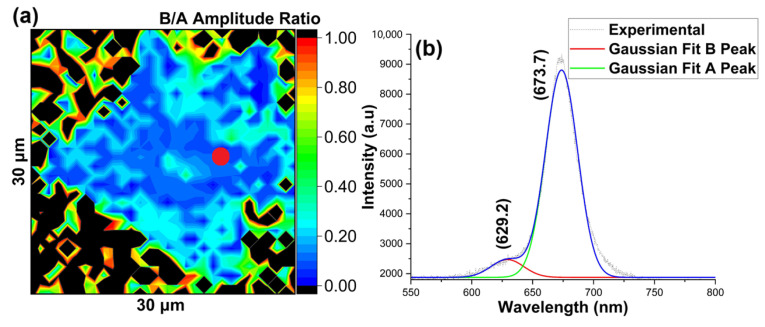
MoS_2_ growth on sapphire substrate (**a**) B/A amplitude ratio map obtained by Gaussian fitting of PL intensity map, (**b**) representative PL spectra of the shown (red) spot. The blue line represents the cumulative fit.

The observed Gaussian fitted B/A amplitude ratio suggested even lower defect density than the as-grown MoS_2_ on SiO_2_/Si substrate, as shown in Figure 9a,b.

**Figure 10:**
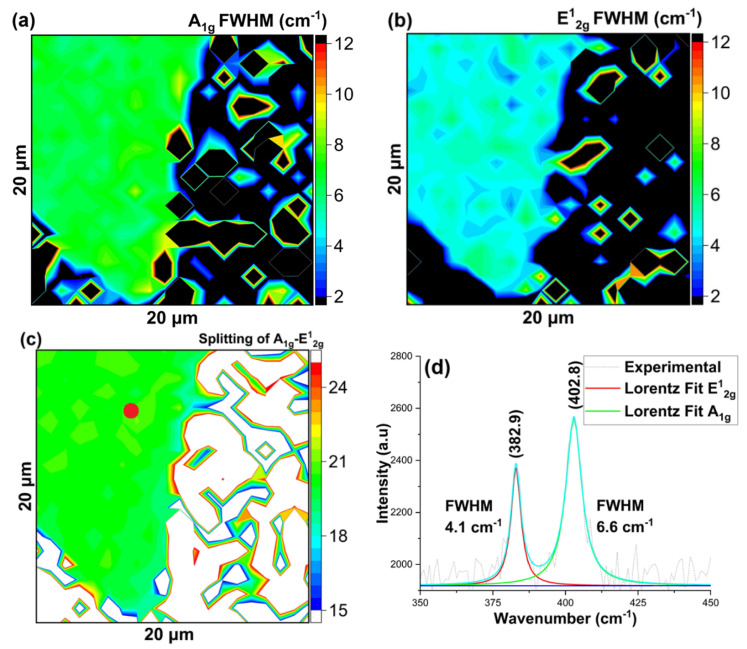
MoS_2_ growth on sapphire Si using quartz boat (**a**) Map of A_1g_ Raman Mode’s Full Width at Half Maxima (FWHM), (**b**) Map of E^1^_2g_ Raman Mode’s Full Width at Half Maxima (FWHM), (**c**) Map of Raman band Splitting of A_1g_ and E^1^_2g_ modes, (**d**) Representative Raman spectra of the indicated (red) spot. The blue line represents the cumulative fit.

**Figure 11:**
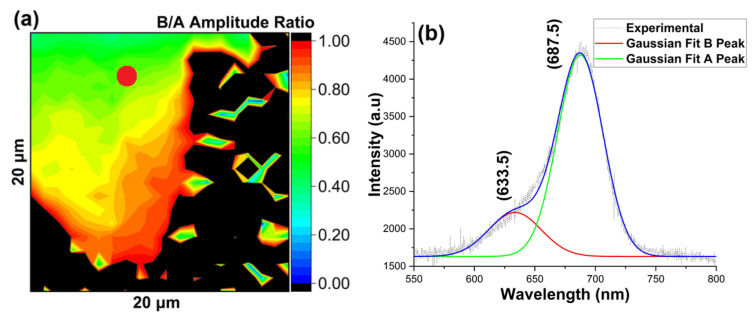
MoS_2_ growth on Si substrate (**a**) B/A amplitude ratio map obtained by Gaussian fitting of PL intensity map, (**b**) representative PL spectra of the shown (red) spot. The blue line represents the cumulative fit.

Lastly, we performed Raman and PL spectra analysis of MoS_2_ grown on a Si substrate with an area of 20 μm × 20 μm of the edge of the MoS_2_ flake grown on a Si substrate, as shown in Figure 10a–d and Figure 11a,b, respectively. Here, the Raman and PL data indicate a defect density similar to the SiO_2_/Si substrate.

For each substrate, the measured PL spectra show intense emissions with well-defined A and B peaks, which are consistent with reported work for monolayer MoS_2_ [25,26]. While our samples are mono- to few-layer MoS_2_, we intentionally performed spectroscopy on optically found monolayer areas to investigate the material quality, which provides a standard measure of the overall quality of the material [24]. The observed PL spectra also confirmed that the analyzed areas correspond to monolayer regions. The PL and Raman maps of MoS_2_ on SiO_2_/Si, sapphire, and Si samples confirmed the excellent quality of the as-grown MoS_2_ on each of these substrates and showed low defect density in the investigated regions [25,26].

To estimate a quantitative measure of the defect densities, we used a linear regression model (R^2^ = 0.71, Figure S5 in the Supplementary Materials) on the reported data of Hossen et al. (2025) [25] and predicted the defect density from the measured FWHM value of our samples, as shown in Table 2. Other researchers have also reported the defect density in the order of 10^12^ for as-grown MoS_2_ monolayer as a high-quality material for applications [34,35]. The B/A intensity ratio of the PL is highly sensitive to non-radiative recombination and well-established qualitative indicator of sample quality. The estimated defect density, along with the observed low B/A ratio, provides evidence that our growth technique yields high crystalline quality of MoS_2_.

The low defect density could be assigned to higher temperature growth as it ensures crystallinity, the adoption of a quartz boat to reduce the carbonaceous impurities, and the desorption of sulfur precursor performed by warming. Also, the low defect density of as-grown MoS_2_ on sapphire could be due to influencing factors like substrate surface energy, lattice mismatch, and chemical interactions. Sapphire substrates have higher surface energy and an ordered surface as compared to the SiO_2_ substrate and enhance uniform nucleation. The lower surface energy, along with the amorphous nature of SiO_2_ substrates, causes bulky growth and promotes defects with grain boundaries [21,36]. Also, the sapphire substrate has a low lattice mismatch (~3.5%) with 2D MoS_2_, and silicon substrates have a higher mismatch. This low lattice mismatch minimizes strain-induced defects such as dislocations or vacancies when MoS_2_ is grown on sapphire. On the other hand, the amorphous nature of silicon substrates promotes polycrystalline growth with relatively higher defect densities [37,38]. In addition, chemical interactions with the substrate influence the quality of the as-grown MoS_2_. On silicon substrates, S-O covalent bonds and potential interdiffusion can increase sulfur vacancies and edge defects [39]. These influencing mechanisms may explain the high-quality growth of MoS_2_ on sapphire substrate in our findings.

## 4. Conclusions

In this research, we investigated a robust, reproducible, potentially scalable, and effective CVD technique for growing mm-scale mono- to few-layer MoS_2_ films by the proximity of the Mo film substrate and multiple target substrates like SiO_2_/Si, Si, and sapphire. We optimized the growth parameters like Mo film thickness, substrate gap, temperature, and sulfur concentration, and studied the effects of using different substrates to achieve consistent MoS_2_ growth. We controlled the contamination and defect density of growth by using a quartz boat instead of a graphite boat. It was found that growth temperature of ~820 °C, and substrate separation of ~0.5 mm proved helpful in producing crystalline, high-quality, and laterally mm-scale, mono- to few-layer MoS_2_ films on target substrates. Raman spectroscopy confirmed the uniformity and high-quality growth, while PL mapping confirmed low defect densities and optical quality of the investigated areas of MoS_2_ growth. This study indicates the potential of unconventional methods for synthesizing MoS_2_, addressing challenges of scalability, substrate versatility, defect density, and high-quality growth of MoS_2_. This work paves the way for future investigations to grow other high-quality TMDs for integration into electronic, optoelectronic, and quantum devices.

## Figures and Tables

**Table 1:** Qualitative and quantitative comparison of proximity techniques.

Precursors /Gas	Temperature (°C)	Growth Time (Minutes)	Flake Size /Type	Substrate	Reference
Mo Film (<100 nm) +S/Ar	~780–860 (Moderate)	~5–8 (Lower)	~1–3 mm (Medium) mono- to few-layer	SiO_2_/Si Sapphire Si	Our Work
Mo Films (20 and 40 nm) +H_2_S	~690–810 (Lower)	~10 (Moderate)	2 inches (Large) monolayer	Sapphire	[14]
MoO_3_ + PTAS + S Bulk MoS_2_ Re-evaporation /Ar	~1000 (Higher)	~120 (Higher)	~10 µm (Small) mono- to few-layer	Sapphire	[15]

**Table 2:** Predicted defect density of the investigated monolayer.

Substrate	FWHM of E^1^_2g_ Mode (cm^−1^)	Predicted Defect Density (cm^−2^)	PL B/A Ratio
SiO_2_/Si	~4–6	1.6–2.7 (×10^12^)	~0.3–0.6
Sapphire	~3–6	0.46–2.7 (×10^12^)	~0.1–0.4
Si	~4–6	1.6–2.7 (×10^12^)	~0.5–0.8

## Data Availability

The raw data supporting the conclusions of this article will be made available by the authors on request.

## Supplementary Materials

The following supporting information can be downloaded at https://www.mdpi.com/article/10.3390/nano16030159/s1, References [24,25,26,40,41,42] are cited in the supplementary materials.
