# Supplementary material for: Proximity-Assisted Synthesis of Large Area MoS2 on Different Target Substrates by Chemical Vapor Deposition Using a Mo Nanofilm Substrate
Source: Nanomaterials (Basel). 2026 Jan 24;16(3):159. doi: 10.3390/nano16030159 (PMC12899469; doi:10.3390/nano16030159)
Supplement: Supplementary file 1 [file nanomaterials-16-00159-s001.zip › nanomaterials-4104310-supplementary.pdf]

Supplementary Information

Proximity-Assisted Synthesis of Large Area MoS_2 on Different Target Substrates by Chemical Vapor Deposition Using a Mo Nanofilm Substrate

Muhammad Tariq [1,2], William Poston [1,2], Norah Aldosari [1,2,3], Gregory Jensen [1,2], Maryam Bizhani [1,2] and Eric Stinaff [1,2,*]

[1] Department of Physics and Astronomy, Ohio University, Athens, OH 45701, USA; mt967721@ohio.edu (M.T.); wp826418@ohio.edu (W.P.); na314617@ohio.edu (N.A.); gj772812@ohio.edu (G.J.); mb795116@ohio.edu (M.B.)
[2] Nanoscale and Quantum Phenomena Institute (NQPI), Athens, OH 45701, USA
[3] Department of Physics and Astronomy, College of Science and Humanities, Prince Sattam bin Abdulaziz University, 173, Al-Kharj 16278, Saudi Arabia
* Correspondence: stinaff@ohio.edu

Mo Film Deposition

The SiO_2/Si multiple substrates were cleaned using acetone and methanol. Molybdenum (Mo) film of thickness less than 100nm was deposited onto SiO_2/Si samples using a Direct Current (DC) sputtering system (Denton Vacuum (DV) 502A). Mo film substrates of different thicknesses were prepared by using a Mo target of 99.95% purity (thickness 6.35 mm, diameter 4-inch, Kurt. J. Leskar). The DC sputtering system was evacuated to a pressure of below 5×10-4 Torr. The argon gas flow rate was controlled and kept at 12 mTorr. A steady current of 200 mA was provided and regulated by a DC power source. Before deposition, the blank sputtering process was conducted for 20-30 minutes. Each substrate was placed on a rotatable substrate carrier stage, which kept the sample isolated from the blank sputtered plasma. The rotatable carrier stage was rotated counterclockwise to set the substrate under the plasma during film deposition. Molybdenum nanofilms of different thicknesses, less than 100nm, were deposited onto SiO_2/Si substrate samples using a DC sputtering system for multiple experiments. A few samples were analyzed for thickness measurement using a scanning electron microscope (SEM). It was revealed that the sputtering system was uncalibrated for a uniform deposition rate. SEM images of the two samples are shown in Figure S1, and using this uncalibrated system, we assumed the average rate of sputtering was 12 to 15 nm/ minute under the given parameters of the sputtering system.

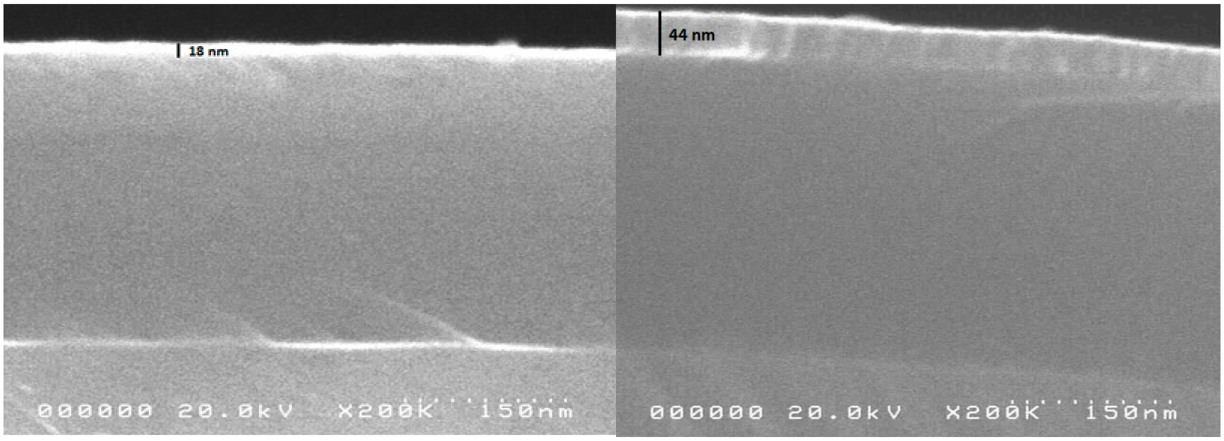

Figure S1: Cross-sectional SEM images (a) showing 18 nm Mo film deposited for 1 minute, (b) showing 44 nm Mo film deposited for 3 minutes.

Annealing of Sapphire Substrate

Commercial sapphire wafers are typically polished to produce at least one flat surface; however, the treatment of the surface of the sapphire sample is important to create a suitable growth template for the growth of 2D materials. Cui et al. (2000) showed that annealing from 1000 °C to 1400 °C for 1 to 3 hours effectively reduces surface roughness to develop terrace-like structures [1]. For the growth of large-area MoS_2 crystals, we used c-plane sapphire wafers with a 1° miscut to a-axis to achieve terrace formation. The sapphire wafer was cut into smaller substrates due to a 1″ diameter constraint in our quartz tube. The individual piece of c-a 1° sapphire substrate was then annealed in a horizontal tube furnace under a flow of Ar/O_2 environment, with ~25 sccm of Ar for 15 min, followed by ~25 sccm of O_2 while keeping the Ar flow. The annealing temperature was ramped to 1,050 °C within 10 minutes and was kept for 4 hours. Finally, the furnace was naturally cooled to room temperature [2].

Distance between the substrates and Growth

In proximity growth, we observed the effect of the distance between the substrates. We performed at distances of ~0.4 millimeters (mm), ~1mm, ~1.3mm, and ~1.7mm as shown in Figure S2. We observed that a small spatial gap of ~0.4 to 1mm favors the growth in terms of size. In the later experiments, we used a gap of ~0.5 mm consistently, as it seemed essential to preserve self-limiting reactions.

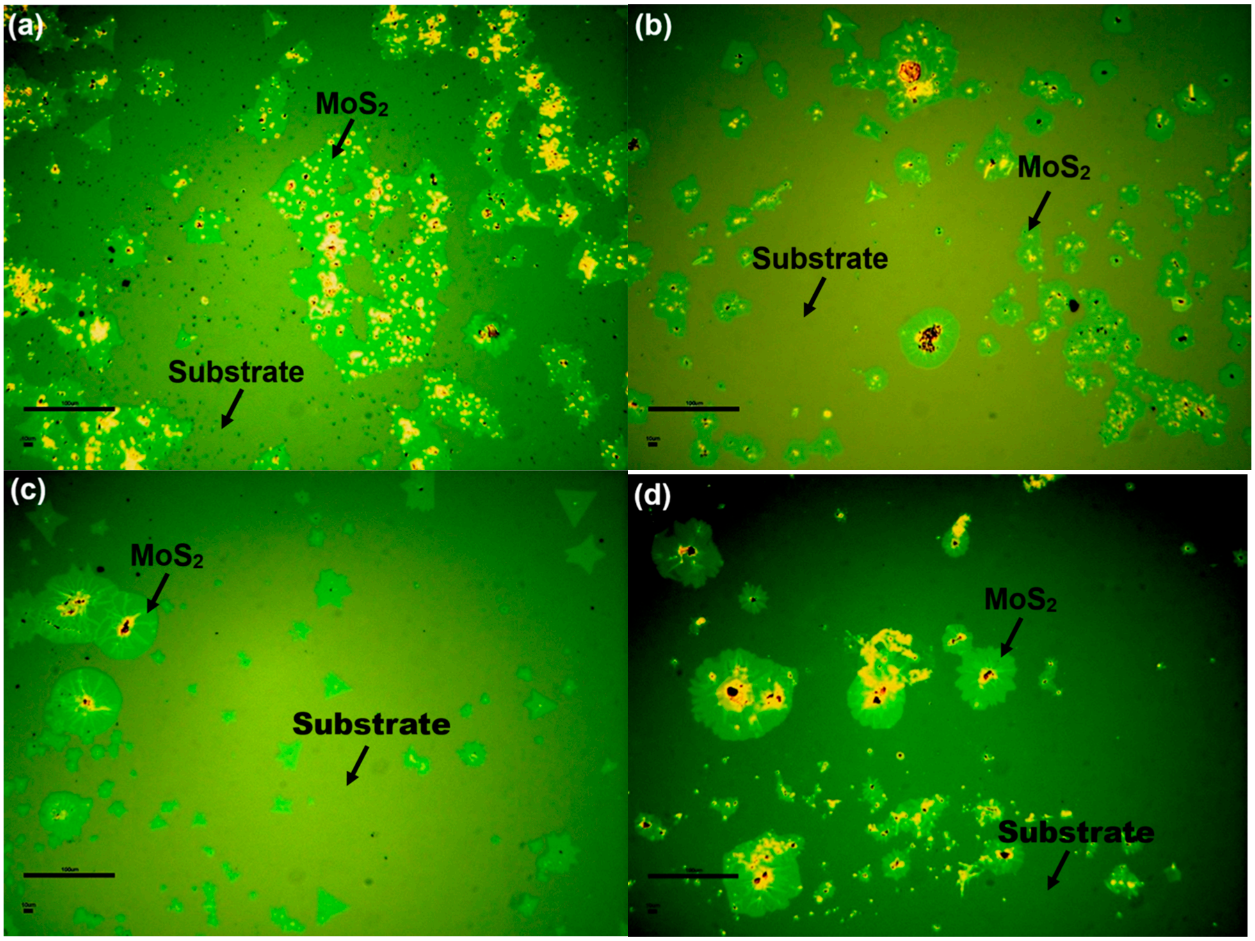

Figure S2. As-grown MoS_2 on SiO_2/Si using a graphite boat and ~40 nm Mo film with gap (a) ~0.4 mm, (b) ~1 mm, (c) ~1.3 mm, and (d) ~1.7 mm (long scale bar at 100 μm), under the same conditions.

Thickness of the Mo film and growth

We sputtered Mo film of different thicknesses by sputtering for 1 minute, 3 minutes, 5 minutes, 8 minutes, and 10 minutes. At lower thickness, we observed no or very small growth due to premature depletion of the film by evaporation, and at higher thickness, the Mo film did not completely oxidize due to the unavailability of residual oxygen in the quartz tube and remained metallic, as observed after the completion of the reaction. A sputtering time between 5 - 8 minutes was found suitable; however, higher thickness also requires a higher temperature for inward oxidation of the Mo film, which could be performed by supplying an oxygen source. A meager growth was observed on either side of the optimal growth parameters. A sputtered film for a duration of 5-8 minutes gives growth on both top and bottom substrates, as shown in Figure S3.

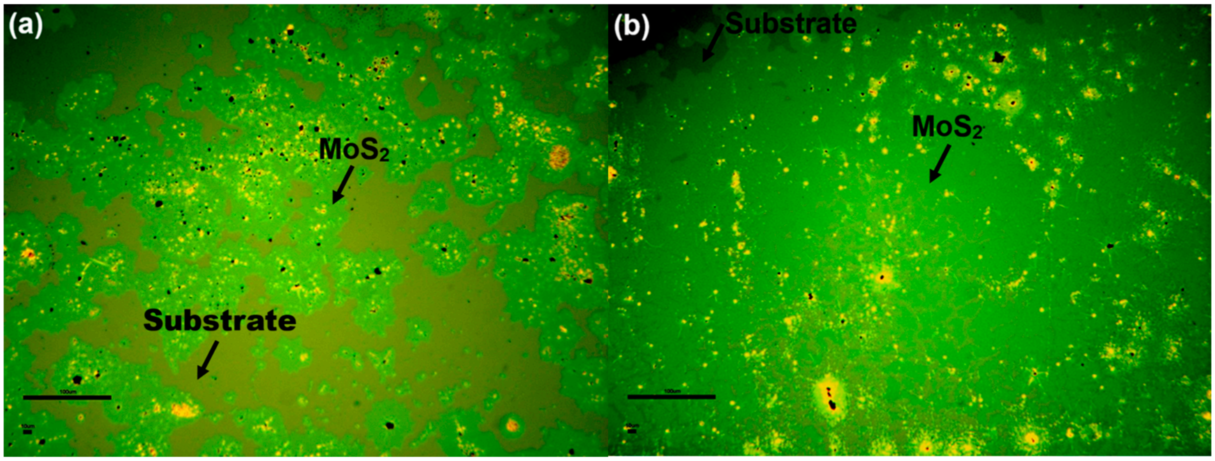

Figure S3. As-grown MoS_2 on SiO_2/Si using a graphite boat (a) ~40 nm Mo film m (b) ~60 nm Mo film (scale bar at 100 μm), under the same conditions.

Raman and PL Spectroscopy of MoS_2 growth on SiO_2/Si Substrate

First, for a monolayer triangular flake grown on SiO_2/Si using a graphite boat, the Lorentz fitting revealed that these vibrational modes are positioned at 381.9 cm^{-1} with FWHM at 10 cm^{-1} and 402.8 cm^{-1} with FWHM at 11.9 cm^{-1}, respectively, with a difference of 20.9 cm^{-1}. The measured Raman spectroscopy results suggest excellent crystallinity comparable to the mechanically exfoliated MoS_2 [3,4]. We conducted PL measurements on the same spot for the same sample, and the spectra supported the monolayer nature of the flake. The Gaussian-fitted PL spectra showed an intense peak at 688.3 nm related to the A exciton of MoS_2, and a low-intensity broad peak at 633.7 nm related to the B exciton of MoS_2, as shown in Figure S4. These measurements were consistent with previously reported results for CVD-developed monolayer MoS_2 [3–6].

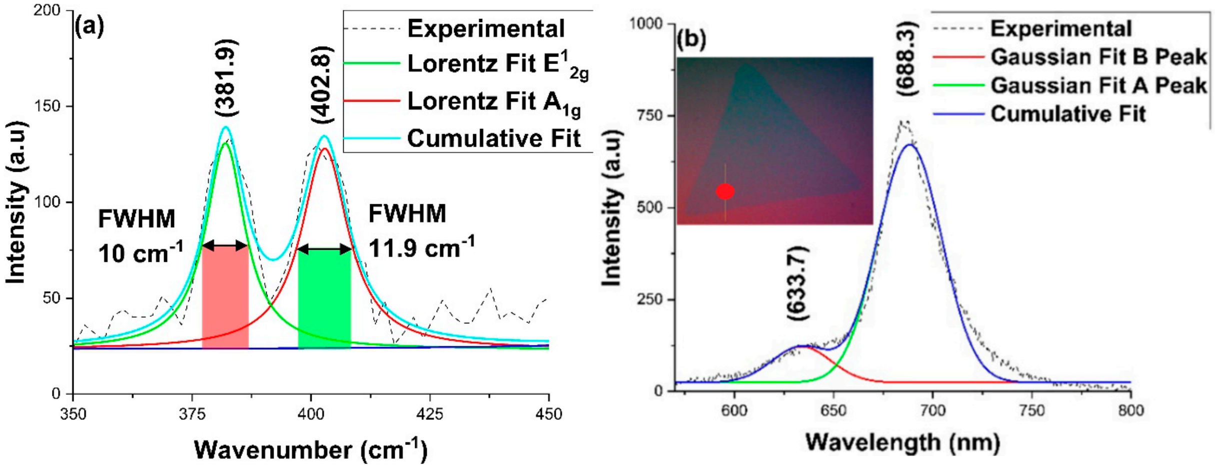

Figure S4. (**a**) Raman spectra of the as-grown MoS_2 monolayer triangular flake using a graphite boat and (**b**) photoluminescence spectra of the same flake.

Linear Regression for Predicting Defect Density

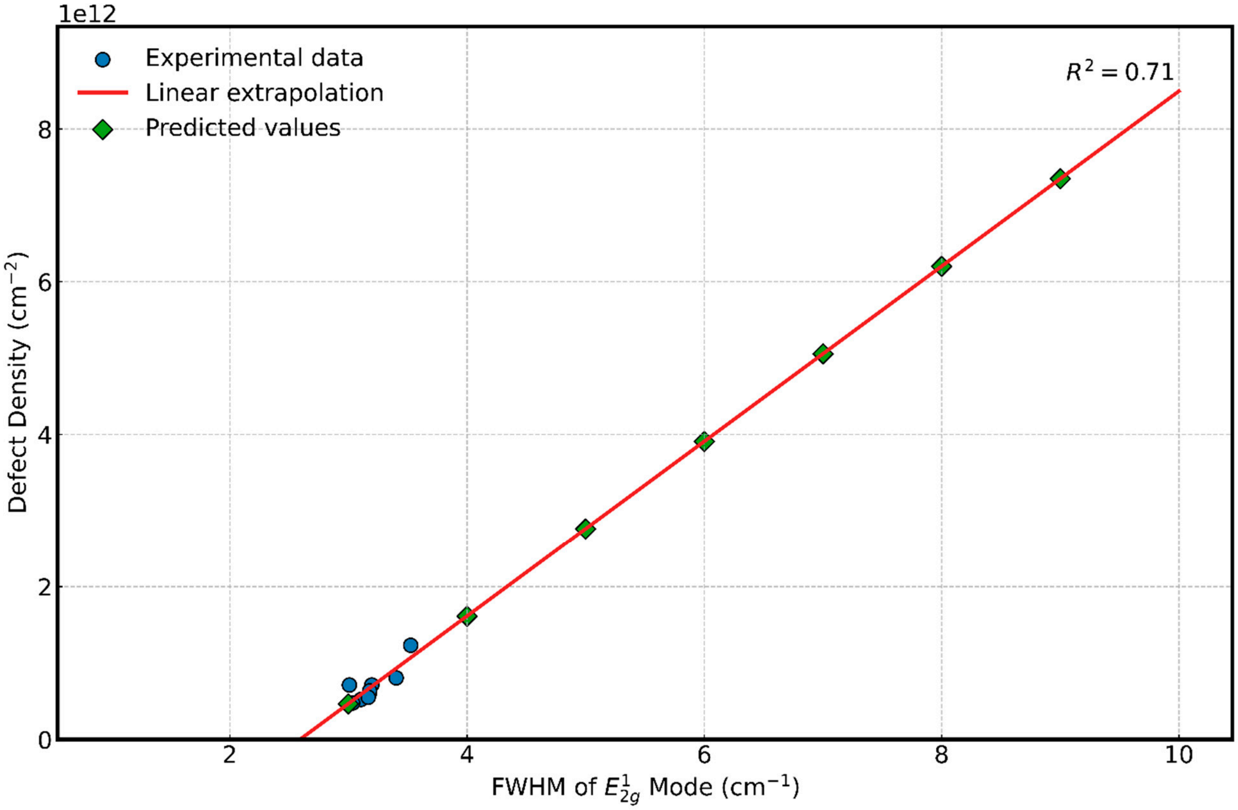

Figure S5. Linear extrapolation for predicting defect density.

```
Linear regression results:
  Slope     = 1.15e+12 cm⁻¹
  Intercept = -2.97e+12 cm⁻²
  R²        = 0.71

Estimated defect densities:
  FWHM = 3.00 cm⁻¹  →  Defect density = 4.66e+11 cm⁻²
  FWHM = 4.00 cm⁻¹  →  Defect density = 1.61e+12 cm⁻²
  FWHM = 5.00 cm⁻¹  →  Defect density = 2.76e+12 cm⁻²
  FWHM = 6.00 cm⁻¹  →  Defect density = 3.91e+12 cm⁻²
  FWHM = 7.00 cm⁻¹  →  Defect density = 5.05e+12 cm⁻²
  FWHM = 8.00 cm⁻¹  →  Defect density = 6.20e+12 cm⁻²
  FWHM = 9.00 cm⁻¹  →  Defect density = 7.35e+12 cm⁻²
```

Millimeter Scale Images

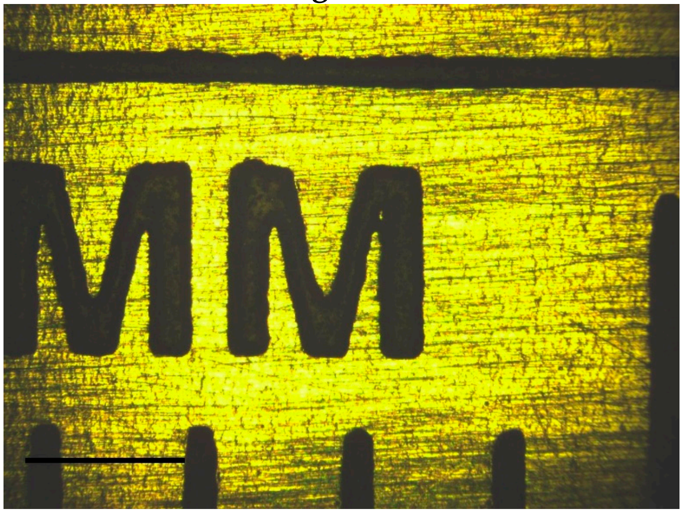

Figure S6. Calibration of the scalebar at 1mm with a ruler.

Sample-1 (Scalebar at 1mm)

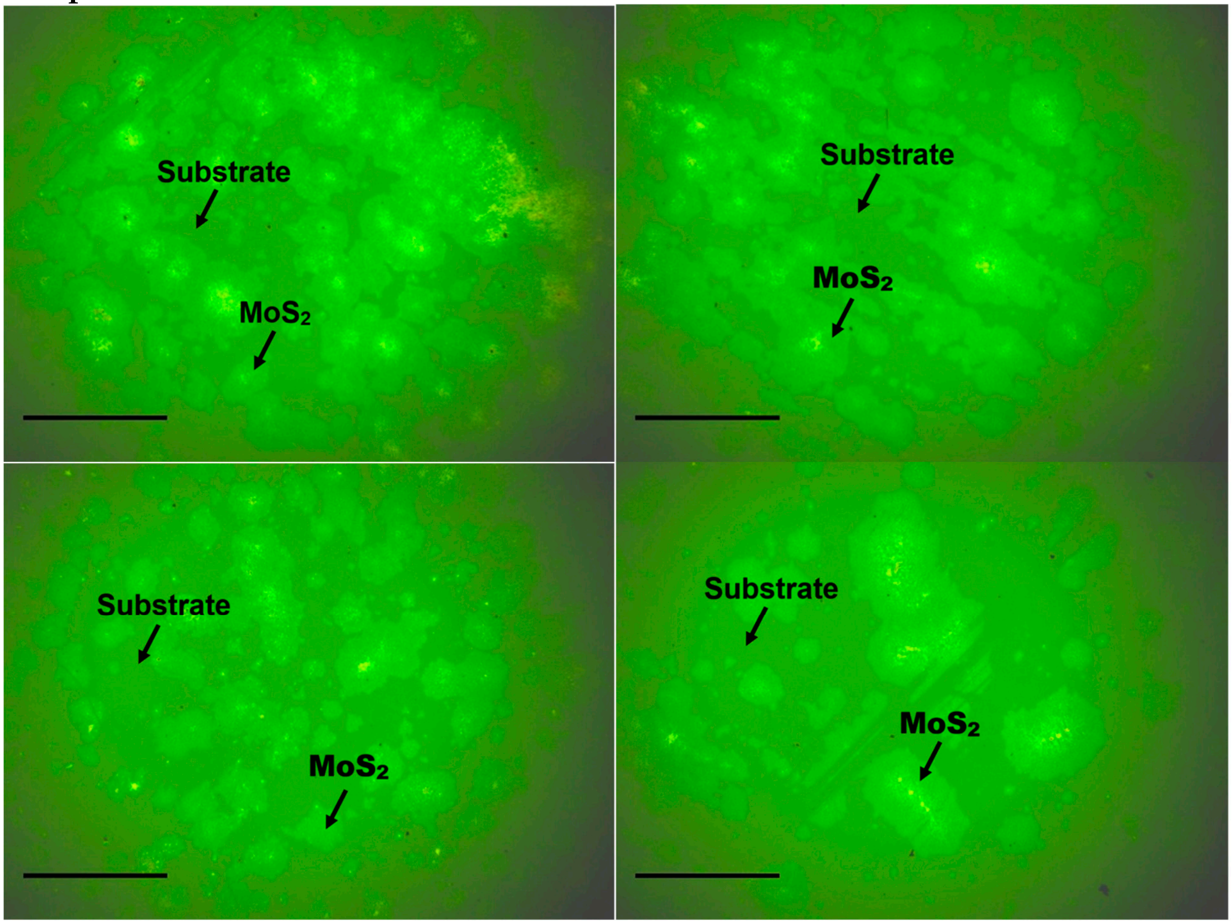

Figure S7. As-grown MoS_2 on SiO_2/Si using a quartz boat with optimal growth parameters.

Sample-2 (Scalebar at 1mm)

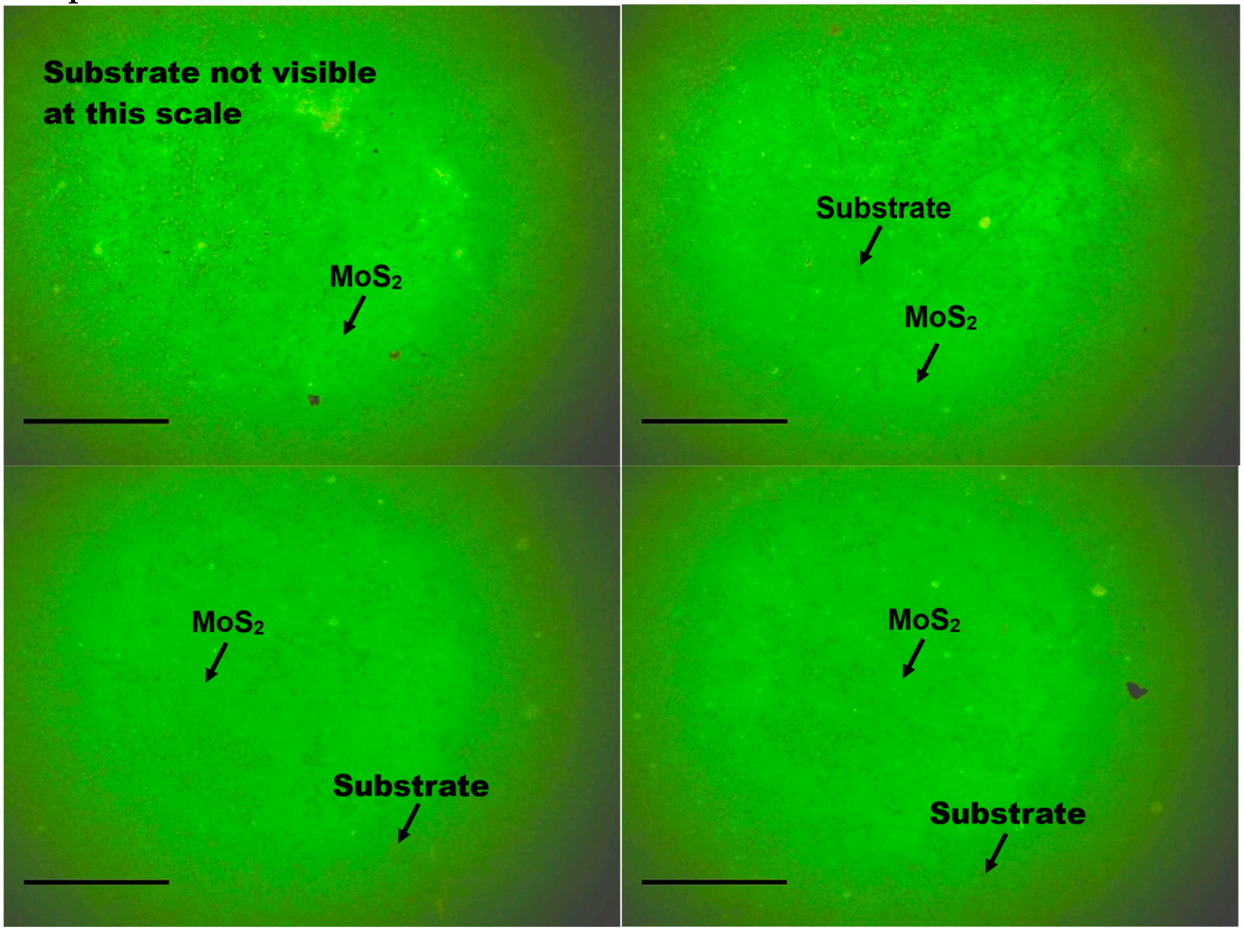

Figure S8. As-grown MoS_2 on SiO_2/Si using a quartz boat with optimal growth parameters.

Sample-3 (Scalebar at 1mm)

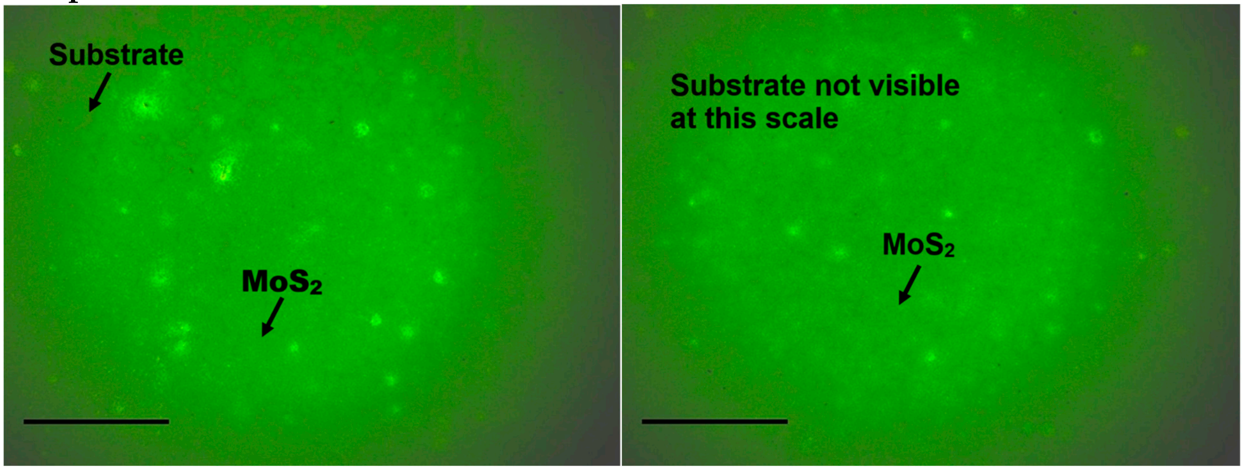

Figure S9. As-grown MoS_2 on SiO_2/Si using a quartz boat with optimal growth parameters.

Sample-4 (Scalebar at 1mm)

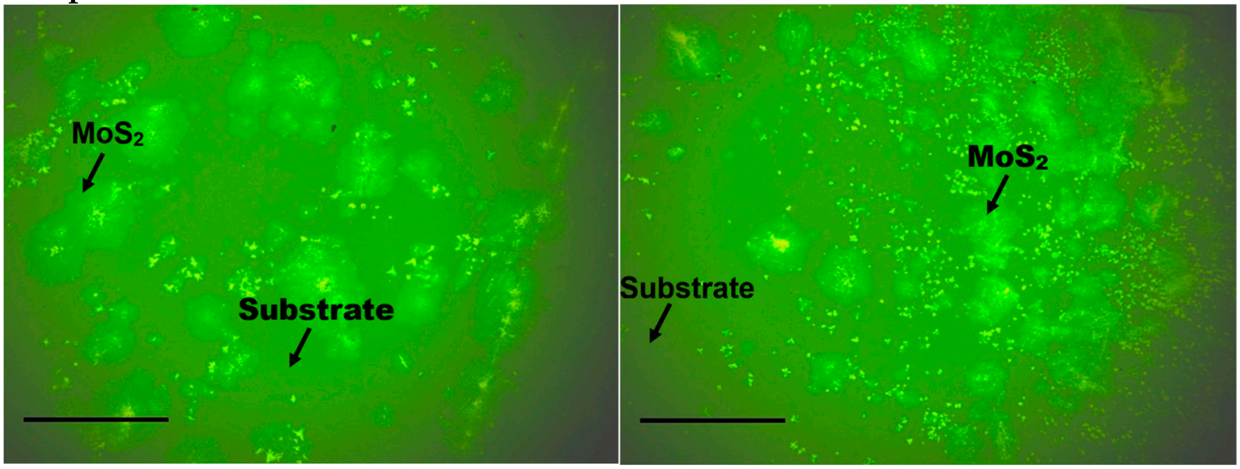

Figure S10. As-grown MoS_2 on SiO_2/Si using a quartz boat with optimal growth parameters.

References

1. Cui, J., Sun, A., Reshichkov, M., Yun, F., Baski, A., & Morkoç, H. (2000). Preparation of sapphire for high quality III-nitride growth. Materials Research Society Internet Journal of Nitride Semiconductor Research, 5(1), e7.
2. Poston, W. B. (2025). Alloying and Structural Modification of Two-Dimensional Transition Metal Dichalcogenides (Doctoral dissertation, Ohio University).
3. Aldosari, N.; Poston, W.; Jensen, G.; Bizhani, M.; Tariq, M.; Stinaff, E. Controlled Oxidation of Metallic Molybdenum Patterns via Joule Heating for Localized MoS2 Growth. *Nanomaterials* **2025**, *15*, 131.
4. Domínguez, A.; Dutt, A.; de Melo, O.; Huerta, L.; Santana, G. Molybdenum oxide 2-D flakes: Role of thickness and annealing treatment on the optoelectronic properties of the material. *J. Mater. Sci.* **2018**, *53*, 6147–6156.
5. Kolobov, A.V.; Tominaga, J. *Two-Dimensional Transition-Metal Dichalcogenides*; Springer: Berlin/Heidelberg, Germany, 2016; Volume 239.
6. Hossen, M.F.; Shendokar, S.; Khan, M.A.R.; Aravamudhan, S. Quantitative Defect Analysis in CVD-Grown Monolayer MoS2 via In-Plane Raman Vibration. *Nano Sel.* **2025**, *6*, e202400103.
